# High virulence sub-populations in *Pseudomonas aeruginosa* long-term cystic fibrosis airway infections

**DOI:** 10.1186/s12866-017-0941-6

**Published:** 2017-02-03

**Authors:** Siobhán O’Brien, David Williams, Joanne L. Fothergill, Steve Paterson, Craig Winstanley, Michael A. Brockhurst

**Affiliations:** 1 Department of Biology, University of York, Wentworth Way, York, YO10 5DD UK; 2 Institute of Infection and Global Health, University of Liverpool, 8 West Derby Street, Liverpool, L69 7B3 UK; 3 Institute of Integrative Biology, University of Liverpool, Crown Street, Liverpool, L69 7ZB UK; 4 Department of Animal and Plant Sciences, University of Sheffield, Sheffield, S10 2TN UK

**Keywords:** *Pseudomonas aeruginosa*, Liverpool epidemic strain, Public goods secretions, Virulence, Cystic fibrosis, Pyoverdine, Pyocyanin, LasA protease

## Abstract

**Background:**

*Pseudomonas aeruginosa* typically displays loss of virulence-associated secretions over the course of chronic cystic fibrosis infections. This has led to the suggestion that virulence is a costly attribute in chronic infections. However, previous reports suggest that overproducing (OP) virulent pathotypes can coexist with non-producing mutants in the CF lung for many years. The consequences of such within-patient phenotypic diversity for the success of this pathogen are not fully understood. Here, we provide in-depth quantification of within-host variation in the production of three virulence associated secretions in the Liverpool cystic fibrosis epidemic strain of *P. aeruginosa*, and investgate the effect of this phenotypic variation on virulence in acute infections of an insect host model.

**Results:**

Within-patient variation was present for all three secretions (pyoverdine, pyocyanin and LasA protease). In two out of three patients sampled, OP isolates coexisted with under-producing mutants. In the third patient, all 39 isolates were under-producers of all three secretions relative to the transmissible ancestor LESB58. Finally, this phenotypic variation translated into variation in virulence in an insect host model.

**Conclusions:**

Within population variation in the production of *P. aeruginosa* virulence-associated secretions can lead to high virulence sub-populations persisting in patients with chronic CF infections.

**Electronic supplementary material:**

The online version of this article (doi:10.1186/s12866-017-0941-6) contains supplementary material, which is available to authorized users.

## Background

Cystic fibrosis (CF) is an autosomal recessive genetic disorder that leads to thickened, viscous mucous obstructing the airways, predisposing patients to lifelong bacterial lung infections. The most prevalent pathogen causing such infections is *Pseudomonas aeruginosa*, which is associated with increased morbidity, a reduction in quality of life and ultimately, mortality in CF patients [1]. Once established in the CF lung, *P. aeruginosa* is often impossible to eradicate [2], owing to high levels of antibiotic resistance and rapid adaptation to a hostile and dynamic host environment [3, 4]. Over the course of chronic CF infections, *P. aeruginosa* undergoes a series of genetic and phenotypic changes in order to adapt to life within the host [5]. These typically include loss of virulence factors such as the quorum-sensing regulated pyocyanin, elastase and protease [6], the siderophore pyoverdine [6], as well as emergence of hypermutators [3], conversion to mucoidity [6, 7] increased antibiotic resistance [8] and the loss of motility [6, 9].

Despite adaptation to the lung environment observed in chronic *P. aeruginosa* CF infections, some highly transmissible strains retain the ability to transmit to new hosts and initiate new infections [10]. For instance, the Liverpool Epidemic Strain (LES) is associated with enhanced morbidity [11], transmission among patients [10], transmission to both non-CF parents of a CF patient [12], and a pet cat [13]. The LES is widespread throughout UK CF clinics [14, 15] and is the most abundant strain of *P. aeruginosa* in the UK CF population [14]. Recently the LES has been reported in the sputa of CF patients in North America [16]. The ability of the LES to maintain transmission, when adaptation to the lung environment is likely to lead to loss of many of the virulence-associated traits thought to be essential for establishing infections is paradoxical, and suggests that more in-depth studies are required to gain a better understanding of phenotypic variation in virulence-associated secretions within the lung.

A growing number of recent studies have identified substantial within-patient phenotypic diversity in *P. aeruginosa* expression of virulence factors, siderophores, quorum sensing, antibiotic resistance, colony morphology, motility and auxotrophy [17–22]. In the LES populations, the phenotypic diversity among isolates within a single CF lung sample is far greater than between samples from the same patient over time, and even between patients [22]. Moreover, patients infected with the LES typically harbour a mixture of OP phenotypes and quorum sensing-defective *lasR* mutants [23]. The persistence of OP phenotypes, in spite of the apparent cost of virulence-associated secretions in chronic CF infections, suggests that these sub-populations may play an important role in the transmissibility of the LES.

Here, we examine within-patient phenotypic diversity in virulence-associated secretions for three CF patients chronically infected with the highly transmissible LES of *P. aeruginosa* for at least 2 years. To characterize within-host variation in three virulence-associated secretions: pyoverdine, pyocyanin and LasA protease, we performed quantitative trait assays on 39 or 40 isolates per patient and the earliest-known LES strain LESB58. Furthermore, we show that within-host phenotypic variation translates into variation in the virulence of acute infections in an insect host model, indicating that population diversity can act as a reservoir for virulent pathotypes.

## Results

### Pyoverdine

**Fig. 1:**
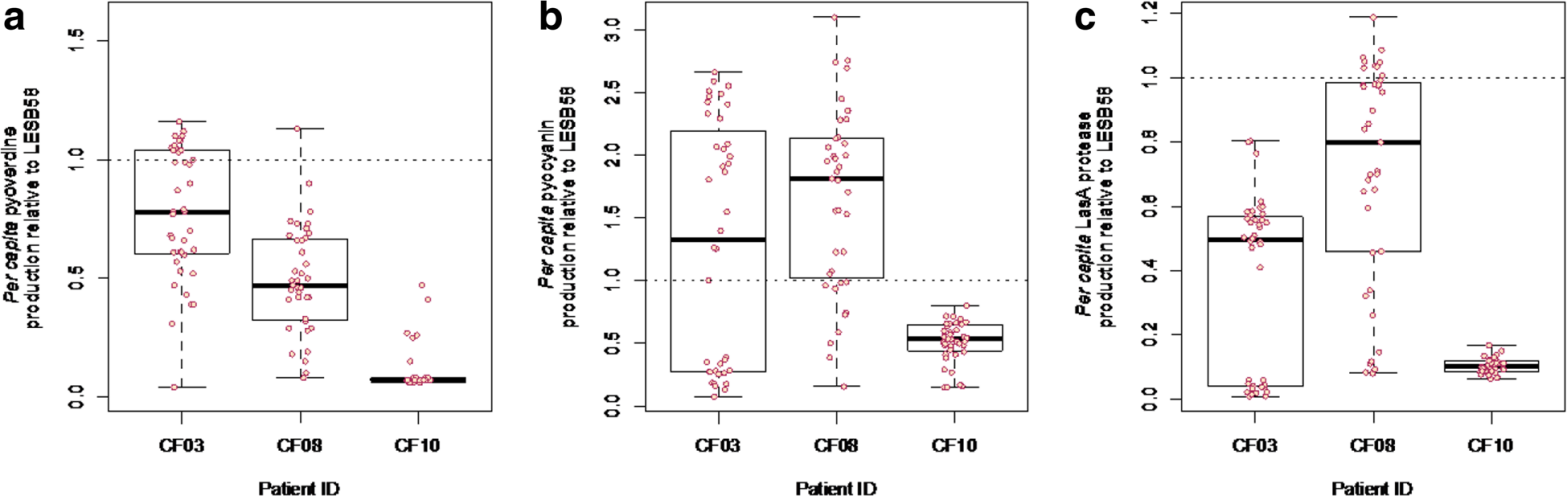
**a** *Per capita* pyoverdine (**b**) pyocyanin and **c** LasA protease production relative to that of LESB58 for each patient (CF03, CF08 and CF10). **a** Mean pyoverdine production is reduced in each patient relative to LESB58 (CF03: V = 129. 5, *p* < 0.001, CF08: t_38_ = 13.916, *p* < 0.001, CF10: V = 0, *p* < 0.001), **b** Mean pyocyanin production is increased relative to LESB58 in patients CF03 and CF08 (CF03: V = 573, *p* < 0.05, CF08: t_38_ = 5.6253, *p* < 0.001), but reduced in CF10 (V = 0, *p* < 0.001), **c** Mean LasA protease is reduced in each patient relative to LESB58 (CF03: V = 0, *p* < 0.001, CF08: V = 101, *p* < 0.001, CF10: t_38_ = 228.18, *p* < 0.001). When *y* = 1, production of the relevant secretion does not differ to that of LESB58. Datapoints represent each of 40 isolates for CF03, 39 for CF08 and 39 for CF10

Each patient harbored populations that, on average, produced less *per capita* pyoverdine relative to LESB58 (1-sample t-/wilcox test against 1; CF03:V = 129. 5, *p* < 0.001, CF08: t_38_ = 13.916, *p* < 0.001, CF10: V = 0, *p* < 0.001, Fig. 1a). Patients CF03 and CF08 showed similar high within-patient variance in pyoverdine production (CF03 range: 4–116%; CF08 range: 8–113%), driven by the coexistence of isolates that produced pyoverdine at levels at least as high as LESB58, with isolates that produced less than 10% relative to LESB58. In contrast, pyoverdine production in patient CF10 was greatly reduced and far less variable among isolates compared to CF03 and CF08, with all isolates producing less than 50% of LESB58’s pyoverdine (CF10 range: 6–47%).

### Pyocyanin

Patients CF03 and CF08 harboured *P. aeruginosa* populations that produced elevated levels of mean *per capita* pyocyanin relative to LESB58 (1-sample t- /wilcox test against 1; CF03: V = 573, *p* < 0.05, CF08: t_38_ = 5.6253, *p* < 0.001, Fig. 1b). However, *per capita* pyocyanin production among isolates displayed high variance within both patients, ranging from 7 to 266% LESB58 levels in CF03, and from 15 to 300% in CF08. In both populations, isolates that produced <20% of the pyocyanin produced by LESB58, coexisted with pyocyanin OP’s (200–300% of LESB58 levels). By contrast, isolates from patient CF10 displayed lower mean *per capita* pyocyanin relative to LESB58 (1-sample Wilcox test against 1; CF10: V = 0, *p* < 0.001, Fig. 1b). Within-population variation in CF10 was much lower than that observed in CF08 or CF03, with pyocyanin production ranging from 14 to 80% that of LESB58 levels.

### LasA protease

Each patient harbored populations that produced significantly lower mean *per capita* protease relative to LESB58 (1-sample t- /wilcox test against 1; CF03: V = 0, *p* < 0.001, CF08: V = 101, *p* < 0.001, CF10: t_38_ = 228.18, *p* < 0.001, Fig. 1c). Patients CF03 and CF08 showed similar high within-host variation in *per capita* protease production (CF03 range: 0–80%; CF08 range: 10–119%), while isolates from patient CF10 showed much reduced levels of protease relative to LESB58, between just 6–16%. In patients CF03 and CF08, therefore, LasA protease underproducers coexisted with isolates producing 80–120% of LESB58 levels, whereas isolates from patient CF10 were all LasA protease underproducers.

### Correlations between different secretions

**Fig. 2:**
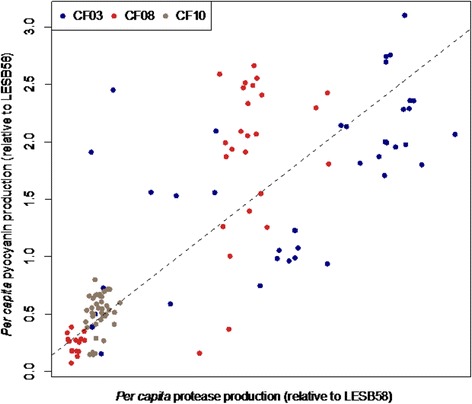
While all three secretions were, overall, positively correlated, we find the strongest relationship between pyocyanin and LasA protease production (both regulated by *LasR*) (X^2^_3_ = 90.761, *p* < 0.0001). Each datapoint represents a single isolate, *n* = 118

We performed principal component analysis on all 118 isolates to investigate associations among production of these secretions. Overall, isolates typically either upregulated all three secretions, or produced very little of any (PC1 explains 73% variance, Additional file 1: Figure S1). While PC1 predicts that all three secretions are positively correlated, pyocyanin and LasA protease show the strongest association. We confirmed this by performing linear mixed effects models (with patient ID as a random variable), which confirmed that the strongest positive correlation was between pyocyanin and protease production (X^2^_3_ = 90.761, *p* < 0.0001, Fig. 2) followed by pyocyanin and pyoverdine (X^2^_3_ = 29.908, *p* < 0.0001) and finally pyoverdine and protease (X^2^_3_ = 19.307, *p* < 0.0001).

We next performed the same analysis independently for each patient (CF03, CF08 and CF10). Isolates from CF03 and CF08 were consistent with findings using the full dataset: all three secretions were positively correlated, with the strongest association between LasA protease and pyocyanin (PC1, CF03: 76.3% variance, Additional file 2: Figure S2; CF08: 65.1% variance, Additional file 3: Figure S3). However isolates from patient CF10 showed a negative correlation between pyocyanin and pyoverdine, so that downregulating pyoverdine was associated with elevated production of pyocyanin (PC1, 62.7% variance, Additional file 4: Figure S4).

### Virulence assays

To assess whether within-patient diversity could act as a resevoir for virulent pathotypes, we performed virulence assays using 40 isolates from the population with the highest phenotypic diversity, CF03, as well as the ancestral strain LESB58. Isolates varied in their ability to form virulent infections in a waxmoth host model, ranging from host death within 1 h of innoculation to completely avirulent infections (no survival difference between infected and control larvae); by comparison, LESB58 killed their waxmoth hosts within an average of 15 h post infection. Rapid mortality of waxmoth hosts was positively associated with pyoverdine production (X^2^_1_ = 19.18, *p* = 0.00001) and, less strongly, with pyocyanin production (X^2^_1_ = 10.072, *p* = 0.0002), but we found no statistically significant association between protease production and mortality rate (X^2^_1_ = 1.18143, *p* > 0.1). Note, however, that pyocyanin and protease production were highly positively correlated in this population, as we have shown above. Hence, on removing the confounding effect of pyocyanin from the full model, protease became an important predictor of waxmoth mortality (X^2^_1_ = 9.2294, *p* = 0.002).

### Lineage specific virulence

**Fig. 3:**
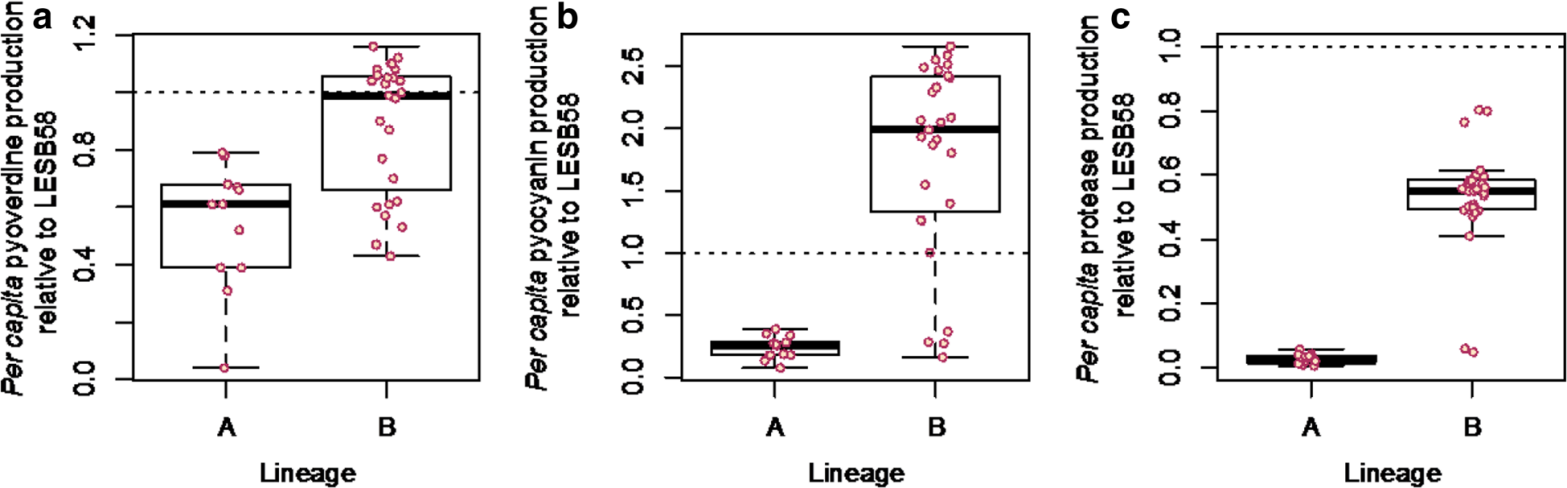
*Per capita* production of (**a**) pyoverdine, **b** pyocyanin and **c** LasA protease relative to LESB58 for patient CF03 only. Secretions were higher for isolates in lineage B compared with lineage A (glm secretion ~ lineage; pyoverdine: F_1,38_ = 18.554, *p* = 0.0001, pyocyanin: F_1,38_ = 50.008, *p* < 0.0001, protease: F_1,38_ = 188.12, *p* < 0.0001). Information on lineages was obtainined from Williams et al. [24]. Each datapoint represents one of 40 isolates originating from patient CF03. When *y* = 1, production of the relevant secretion does not differ to that of LESB58

Williams et al. [24] analysed the phylogenetic relationship between the 40 *P. aeruginosa* isolates from patient CF03 based on whole genome sequences for each isolate. This population comprises of two LES lineages, A and B. Lineage A is characterised by a non-synonymous mutation in the *lasR* gene, and so we hypothesised that the production of pyocyanin and protease (which are LasR dependent), and to a lesser extent, pyoverdine (which is upregulated by, but not dependent on LasR) would show an association with lineage. Production of all three secretions was higher for isolates in lineage B compared with lineage A (glm secretion ~ lineage; pyoverdine: F_1,38_ = 18.554, *p* = 0.0001, pyocyanin: F_1,38_ = 50.008, *p* < 0.0001, protease: F_1,38_ = 188.12, *p* < 0.0001, Fig. 3). Accordingly, waxmoths infected with isolates from lineage B (with intact *lasR*) were killed 3 times faster than those infected with isolates from lineage A (z = 6.39, *p* < 0.0001).

## Discussion

The secretion of virulence-factors by *P. aeruginosa* is thought to be associated with the establishment of infection in CF. The current paradigm is that if these acute infections become chronic, virulence-associated secretions become less beneficial or costly, and are lost *via* the accumulation of mutations, particularly in global regulators (e.g. *lasR*) [6, 25]. By quantifying the production of three virulence-associated secretions (pyocyanin, LasA protease and pyoverdine) for 118 *P. aeruginosa* isolates from three CF patients, we show that the loss of virulence-associated secretions is not always universal within patients. We report that in two out of three patients, isolates that secreted very little (underproducers), coexisted with isolates that produced secretions at levels at least as high, or several fold higher (overproducers), than the earliest-known LES strain LESB58. Moreover, we show that this diversity has consequences for virulence: overproducing isolates displayed higher virulence in a waxmoth larval host, suggesting that within population diversity acts as a ‘resevoir’ for high-virulence sub-populations even in long-term chronic infections. Hence, while the general paradigm is that population-level production of virulence-associated secretions decline during chronic infection [6, 25], this does not imply that the ability to cause virulent infections is completely lost. In light of this, effectively diagnosing *P. aeruginosa* CF infections strongly depends on in-depth microbiological screening that takes into account within-patient diversity.

Our study reveals that overall, within-host *P. aeruginosa* isolates exhibit reduced population-level production of pyoverdine and LasA protease relative to LESB58. Since we sacrifice sampling at the patient level at the expense of more in-depth analysis of within-patient diversity, we cannot make any firm claims about whether this finding is representative of *P. aeruginosa* chronic infections in general. However, our findings are consistent with previous studies that sample fewer isolates from a larger number of patients, such as Andersen et al. [26] (13 isolates/ patient sample) and Hoffman et al. [27] (2.8 isolates/ patient sample). Our results also reveal differences between populations. Notably, while infections within patients CF03 and CF08 demonstrated high within-population diversity, whereby over- and under-producing isolates coexisted, isolates from patient CF10 tended to produce very little, if any, of the three secretions that we quantified. This finding is supported by previous work that investigated the phylogenetic relationship between isolates within these three patients, showing that while CF03 and CF08 populations consist of two genetically diverged lineages, the CF10 population consists of just one [24]. Our finding that patient CF10 harboured less phenotypic diversity than CF03 and CF08 may be partially explained by duration of infection. At the time of sampling, patient CF10 had been infected with *P. aeruginosa* for the least amount of time (2 years), compared with CF03 (3 years) and CF08 (>5 years) [22]. However, our finding that CF03 and CF08 show similar levels of diversity, when CF03 and CF10 have been infected for more similar periods of time suggests that duration of infection is not the only predictor of phenotypic diversity.

This study, along with several others [23, 28] suggests that OP phenotypes are commonly found in combination with underproducers within the CF lung. While the explanation for the maintenance of OP phenotypes in chronic infections remains to be elucidated, this study provides a potential explanation: OP phenotypes may retain the ability to transmit between patients, and levels of secreted virulence-factor production may therefore represent a trade-off between adaptation to either a chronic or acute infection lifestyle. However, evolution has no foresight and so it would be difficult to see how maintaining virulence in a chronic context would be favoured, even if a new infection could be better initiated in the future. Hence, while highly virulent subpopulations may be beneficial for transmission, it is likely that other factors play a role in shaping virulence in chronic infections. One possibility is that secretions that confer a benefit to nearby conspecifics will be lost from a population, because non-producing conspecifics can still access the benefits without paying the cost of making the secretion. Hence, non-producing ‘cheats’ invade populations of ‘cooperative’ producers [26]. Moreover, recent in vitro work has shown that coevolution between *P. aeruginosa* cooperators and cheats enables them to coexist, because cooperators become more difficult to exploit, and cheats become more efficient exploiters [29]. While the exploitation of virulence-associated secretions by non-producers could explain the emergence of non-producing LasR mutants in the context of CF, this has yet to be experimentally investigated.

Understanding the bacterial characteristics that contribute most to disease is a major path toward developing novel “disarming” antimicrobials [30]. A handful of previous studies have monitored the ability of phenotypically varied LES isolates to form acute infections. In a mouse model of acute infections, Carter et al. [31] revealed subtle variation in four LES subtypes (including LESB58) in their ability to kill their host. LES431, (associated with reduced biofilm, and elevated pyocyanin, elastase and protease production) was best at forming acute infections in a mouse model whereas all mice infected with the quorum-sensing-negative *lasR* mutant LES400 survived the entire duration of the experiment. While this might suggest that quorum-sensing is important for establishing acute infection in mice, all LESB58 (with an intact *lasR* system) infected mice also survived. Similar results were observed in fruit flies: LES431 and LES400 formed the most and least virulent infections, respectively, out of seven LES strains from different sources of infection [32]. Our study reveals that the production of pyoverdine, pyocyanin and LasA protease are all important predictors of virulence in a waxmoth larva host. While these secretions were highly correlated, pyoverdine emerged as the greatest contributor to virulence. A recent meta-analysis has shown that in lab strains such as PAO1 and PA14, the effect of pyoverdine, albeit frequently contributing to disease, is relatively minor, and varies considerably across infection models [33]. While these strains were initially isolated from clinical settings, they have subsequently undergone evolution in the laboratory environment [34], and hence may differ substantially from the clinical strains used in this study. Hence, the role of pyoverdine may be more prominent in studies such as this one that use clinical populations comprising of OP strains and *lasR* mutants. Our results suggest that to fully understand the ability of virulence-associated secretions to reduce host health and survival, it is vital to carry out additional studies on diverse clinical isolates. This approach is especially relevant for developing novel therapeutics that curb *P. aeruginosa* pathogenicity by targeting a specific virulence-associated secretion.

## Conclusion

Isolates of a CF epidemic strain of *P. aeruginosa* have been previously shown to display a) loss of viurlence-encoding genes over the course of infection and b) considerable within-patient phenotypic diversity. We demonstrate that this diversity in virulence-associated secretions among isolates means that OP virulent strains can sometimes be present in combination with non-virulent strains. Hence, despite the common loss of virulence as *P. aeruginosa* adapts to the CF lung environment, the persistence of high-virulence subpopulations may allow high rates of transmission among patients to be retained.

## Methods

### Patients and samples

Routine sputum samples from CF patients harboring long-term LES infections were collected from 10 patients between January 2009 and May 2011 [22, 28]. Briefly, ~40 colonies were isolated per sample, ensuring that each different colony morphology type was proportionally represented. Each isolate was confirmed as *P. aeruginosa* LES, using polymerase chain reaction assays. For this study, we focused on patients CF03, CF08 and CF10, because they were best matched in terms of patient characteristics (all female, aged between 20 and 25, FEV_1_ = 36–37%). A single sputum sample from each patient, collected in January 2009, was selected for our experimental investigations (CF03 (Stable_2); CF08 (Stable_1); CF10 (Stable_1), as detailed in [22]). All samples used were isolated during routine visits to the clinic (i.e. lung condition was relatively stable). Population genetic structure for each sample is detailed in Williams et al. (2015), revealing that two of our samples (CF08 and CF08) comprise of two lineages, while CF10 consists of a single lineage. Due to difficulties in culturing some isolates, our dataset comprised of 40 isolates from CF03, 39 isolates from CF08 and 39 isolates from CF10. As a comparison we used LESB58 [35], the earliest archived LES isolate.

### Pyoverdine measurements

We quantified *per capita* production of the most costly and efficient iron-chelating siderophore, pyoverdine [36–38] using a pyoverdine specific exitation-emmission assay [38–41]. 118 CF isolates, and LESB58 were cultured in 6 ml Lysogeny broth (LB) for 24 h at 37 °C, shaken at 180 rpm. The optical density (OD; A_600_) was measured at 600 nm and standardized to 1 (~10^9^ CFU ml^−1^) using M9 buffer. 10^7^ cells were then added to 200 μl Casamino acid (CAA) medium in 96 well-plates, made iron-limited by the addition of freshly made filter-sterilised 100 μg/ml human apotransferrin and 20 mM NaHCO_3_ immediately before use. Since siderophore production is repressed when there is an excess of Fe^2+^ [42, 43], iron-limitation ensures that siderophores are essential for growth and stimulates their production. Outer wells were filled with 200 μl CAA media only to minimise evaporation. Plates were grown static at 37 °C for 24 h. Cultures were then diluted ×10 with ddH_2_0 and fluorescence of each culture was measured at 460 nm following excitation at 400 nm, using a Tecan infinite M200 pro spectrophotometer. OD was measured at 600 nm, and the ratio fluorescence/OD was employed as a quantitative measure of *per capita* pyoverdine production [44, 45].

### Pyocyanin measurements

*Per capita* pyocyanin production was determied for each CF isolate and LESB58. Isolates were cultured in 6 ml LB broth for 24 h at 37 °C, shaken at 180 rpm. OD was standardized to 1 (~10^9^ CFU ml^−1^), using M9 buffer. 10^7^ cells were then innoculated into 6 ml LB broth, and grown at 37 °C for 24 h shaken at 180 rpm. 1 ml of each culture was centrifuged at 18,000 g for 3 minutes. *Per capita* pyocyanin production was measured for each isolate by measuring A_691_ of the supernatant, and then standardizing by bacterial OD [46].

### LasA protease measurements

LasA protease activity was quantified by determining the ability of *P. aeruginosa* culture supernatants to lyse boiled *Staphyloccous aureus* cells [47]. Briefly, a 20 ml volume of overnight *S. aureus* cells was boiled for 10 min in a waterbath, and then centrifuged for 10 min at 10,000 rpm. The resulting pellet was resuspended in 0.02 M aTris-HCl (ph 8.5) to an OD of ~ 0.9. Each bacterial isolate was cultured in 6 ml LB broth for 24 h at 37 °C, shaken at 180 rpm, and OD standardized to 1 (~10^9^ CFU ml^−1^) using M9 buffer. 10^7^ cells were then added to a further 6mls LB broth, and grown at 37 °C for 24 h shaken at 180 rpm. Supernatant was obtained by centrifuging 1 ml at 18,000 g for 3 min. 7.5 μl of each LES supernatant was mixed with 142.5 μl *S. aureus* cells and OD was measured at time 0 and again after 60 min. This assay was replicated three times for each isolate. *Per capita* LasA protease activity was quantified by dividing the reduction in OD for each isolate by the OD of each starting population.

### In vivo virulence assays

In order to investigate whether phenotypic diversity could translate into virulence diversity, we performed a survival analysis using 40 isolates from patient CF03 only. This patient was chosen because 1) these isolates all have complete genome sequences available, so survival data would be a valuable asset to this dataset and 2) CF03 exhibited a high amount of within-host variation. Forty isolates from CF03, and LESB58 were cultured in 6 ml LB broth for 24 h at 37 °C, shaken at 180 rpm. OD was standardized to 0.1 (~10^8^ CFU ml^−1^), using sterile NaCl (80% w/v). Larvae were purchased the day prior to inoculation from Pets at Home, York, UK. Larvae were swabbed with 70% v/v ethanol to prevent contamination of the injection site, and diluted isolates were injected in the abdomen using a Hamilton syringe. The injection volume was 7 μl in all cases (~10^6^ cells). Six larvae were assigned to each of 41 treatments (40 LES isolates from CF03 and LESB58). A further 6 larvae were injected with 7 μl 80% NaCl as negative controls; their mortality rate was null. Larvae were incubated at 37 °C and monitored for death at hourly intervals between 0 and 24 h post-inoculation. Larvae were scored as dead if they failed to respond to mechanical stimulation of the head.

### Statistical analysis

All measurements were standardised by bacterial density to obtain *per capita* values.

Assay measurements were divided by the relevant LESB58 value, to determine the change in their production relative to the ancestor. When this value =1 evolved isolates and LESB58 did not differ in quantity of secreted product. To assess differences we performed one-sample t-tests/wilcox tests against 1. General linear models were employed to assess whether lineage predicted levels of production of each secretion in patient CF03. We used three linear mixed effects models, to verify the relationship between pyocyanin and pyoverdine, pyocyanin and LasA protease, and pyoverdine and lasA protease, assigning “patient” as a random effect. Survival analysis was performed using a cox proportional hazards model with three continuous variables (pyocyanin, pyoverdine and protease) as the response variable, including 3- way and 2-way interactions. All data were analysed using R version 2.15.1 [48].

## Additional files

Additional file 1: Figure S1. Results of principal component analysis whereby all 188 isolates were pooled to investigate associations among production of these secretions. Overall, isolates typically either upregulated all three secretions, or produced very little of any (PC1 explains 73% variance). (GIF 368 kb)
Additional file 2: Figure S2. Results of principal component analysis using phenotypic measurements from 40 isolates originating from patient CF03, showing that all three secretions were positively correlated, with the strongest association between LasA protease and pyocyanin (PC1, 76.3% variance). (GIF 194 kb)
Additional file 3: Figure S3. Results of principal component analysis using phenotypic measurements from 39 isolates originating from patient CF08, showing that all three secretions were positively correlated, with the strongest association between LasA protease and pyocyanin (PC1, 65.1% variance). (GIF 212 kb)
Additional file 4: Figure S4. Results of principal component analysis using phenotypic measurements from 39 isolates originating from patient CF10, revealing a negative correlation between pyocyanin and pyoverdine (PC1, 62.7% variance). (GIF 79 kb)

## Abbreviations

CAA: Casamino acid
CF: Cystic fibrosis
LES: Liverpool epidemic strain
OD: Optical density
OP Isolate: Isolate over-producing virulence associated secretions

## References

[1] Pressler T, Bohmova C, Conway S, Dumcius S, Hjelte L, Høiby N (2011). Chronic Pseudomonas aeruginosa infection definition: EuroCareCF Working Group report. J Cyst Fibros.

[2] Hart CA, Winstanley C (2002). Persistent and aggressive bacteria in the lungs of cystic fibrosis children. Br Med Bull.

[3] Oliver A, Cantón R, Campo P, Baquero F, Blázquez J (2000). High frequency of hypermutable pseudomonas aeruginosa in cystic fibrosis lung infection. Science.

[4] Winstanley C, O’Brien S, Brockhurst MA (2016). Pseudomonas aeruginosa evolutionary adaptation and diversification in cystic fibrosis chronic lung infections. Trends Microbiol.

[5] Folkesson A, Jelsbak L, Yang L, Johansen HK, Ciofu O, Høiby N (2012). Adaptation of pseudomonas aeruginosa to the cystic fibrosis airway: an evolutionary perspective. Nat Rev Microbiol.

[6] Smith EE, Buckley DG, Wu Z, Saenphimmachak C, Hoffman LR, D’Argenio DA (2006). Genetic adaptation by pseudomonas aeruginosa to the airways of cystic fibrosis patients. Proc Natl Acad Sci U S A.

[7] Govan JR, Deretic V (1996). Microbial pathogenesis in cystic fibrosis: mucoid pseudomonas aeruginosa and burkholderia cepacia. Microbiol Rev.

[8] Breidenstein EBM, de la Fuente-Núñez C, Hancock REW (2011). Pseudomonas aeruginosa: all roads lead to resistance. Trends Microbiol.

[9] Mahenthiralingam E, Campbell ME, Speert DP (1994). Nonmotility and phagocytic resistance of pseudomonas aeruginosa isolates from chronically colonized patients with cystic fibrosis. Infect Immun.

[10] Fothergill JL, Walshaw MJ, Winstanley C (2012). Transmissible strains of pseudomonas aeruginosa in cystic fibrosis lung infections. Eur Respir J.

[11] Al-Aloul M, Crawley J, Winstanley C, Hart CA, Ledson MJ, Walshaw MJ (2004). Increased morbidity associated with chronic infection by an epidemic pseudomonas aeruginosa strain in CF patients. Thorax.

[12] McCallum SJ, Gallagher MJ, Corkill JE, Hart CA, Ledson MJ, Walshaw MJ (2002). Spread of an epidemic pseudomonas aeruginosa strain from a patient with cystic fibrosis (CF) to non-CF relatives. Thorax.

[13] Mohan K, Fothergill JL, Storrar J, Ledson MJ, Winstanley C, Walshaw MJ (2008). Transmission of pseudomonas aeruginosa epidemic strain from a patient with cystic fibrosis to a pet cat. Thorax.

[14] Scott FW, Pitt TL (2004). Identification and characterization of transmissible pseudomonas aeruginosa strains in cystic fibrosis patients in England and Wales. J Med Microbiol.

[15] Edenborough FP, Stone HR, Kelly SJ, Zadik P, Doherty CJ, Govan JRW (2004). Genotyping of pseudomonas aeruginosa in cystic fibrosis suggests need for segregation. J Cyst Fibros.

[16] Aaron SD, Vandemheen KL, Ramotar K, Giesbrecht-Lewis T, Tullis E, Freitag A (2010). Infection with transmissible strains of Pseudomonas aeruginosa and clinical outcomes in adults with cystic fibrosis. JAMA J Am Med Assoc.

[17] Workentine ML, Sibley CD, Glezerson B, Purighalla S, Norgaard-Gron JC, Parkins MD (2013). Phenotypic heterogeneity of pseudomonas aeruginosa populations in a cystic fibrosis patient. Plos One.

[18] Darch SE, Mcnally A, Harrison F, Corander J, Barr HL, Paszkiewicz K (2015). Recombination is a key driver of genomic and phenotypic diversity in a pseudomonas aeruginosa population during cystic fibrosis infection. Sci Rep.

[19] Ashish A, Paterson S, Mowat E, Fothergill JL, Walshaw MJ, Winstanley C (2013). Extensive diversification is a common feature of pseudomonas aeruginosa populations during respiratory infections in cystic fibrosis. J Cyst Fibros.

[20] Clark ST, Diaz Caballero J, Cheang M, Coburn B, Wang PW, Donaldson SL (2015). Phenotypic diversity within a pseudomonas aeruginosa population infecting an adult with cystic fibrosis. Sci Rep.

[21] Wilder CN, Allada G, Schuster M (2009). Instantaneous within-patient diversity of pseudomonas aeruginosa quorum-sensing populations from cystic fibrosis lung infections. Infect Immun.

[22] Mowat E, Paterson S, Fothergill JL, Wright EA, Ledson MJ, Walshaw MJ (2011). Pseudomonas aeruginosa population diversity and turnover in cystic fibrosis chronic infections. Am J Respir Crit Care Med.

[23] Fothergill JL, Panagea S, Hart CA, Walshaw MJ, Pitt TL, Winstanley C (2007). Widespread pyocyanin over-production among isolates of a cystic fibrosis epidemic strain. BMC Microbiol.

[24] Williams D, Evans B, Haldenby S, Walshaw MJ, Brockhurst MA, Winstanley C (2015). Divergent, coexisting pseudomonas aeruginosa lineages in chronic cystic fibrosis lung infections. Am J Respir Crit Care Med.

[25] Nguyen D, Singh PK (2006). Evolving stealth: genetic adaptation of pseudomonas aeruginosa during cystic fibrosis infections. Proc Natl Acad Sci U S A.

[26] Andersen SB, Marvig RL, Molin S, Krogh H, Griffin AS (2015). Long-term social dynamics drive loss of function in pathogenic bacteria. Proc Natl Acad Sci U S A.

[27] Hoffman LR, Kulasekara HD, Emerson J, Houston LS, Burns JL, Ramsey BW (2009). Pseudomonas aeruginosa lasR mutants are associated with cystic fibrosis lung disease progression. J Cyst Fibros.

[28] Fothergill JL, Mowat E, Ledson MJ, Walshaw MJ, Winstanley C (2010). Fluctuations in phenotypes and genotypes within populations of pseudomonas aeruginosa in the cystic fibrosis lung during pulmonary exacerbations. J Med Microbiol.

[29] Kümmerli R, Santorelli L a, Granato ET, Dumas Z, Dobay a, Griffin a S (2015). Co-evolutionary dynamics between public good producers and cheats in the bacterium Pseudomonas aeruginosa. J Evol Biol.

[30] Clatworthy AE, Pierson E, Hung DT (2007). Targeting virulence: a new paradigm for antimicrobial therapy. Nat Chem Biol.

[31] Carter MEK, Fothergill JL, Walshaw MJ, Rajakumar K, Kadioglu A, Winstanley C (2010). A subtype of a pseudomonas aeruginosa cystic fibrosis epidemic strain exhibits enhanced virulence in a murine model of acute respiratory infection. J Infect Dis.

[32] Salunkhe P, Smart CHM, Morgan JAW, Panagea S, Walshaw MJ, Hart CA (2005). A cystic fibrosis epidemic strain of pseudomonas aeruginosa displays enhanced virulence and antimicrobial resistance. J Bacteriol.

[33] Granato ET, Harrison F, Kümmerli R, Ross-Gillespie A (2016). Do Bacterial “Virulence Factors” Always Increase Virulence?. A Meta-Analysis of Pyoverdine Production in Pseudomonas aeruginosa As a Test Case. Front. Microbiol..

[34] Klockgether J, Cramer N, Wiehlmann L, Davenport CF, Tummler B (2011). Pseudomonas aeruginosa genomic structure and diversity. Front Microbiol.

[35] Winstanley C, Langille MGI, Fothergill JL, Kukavica-Ibrulj I, Paradis-Bleau C, Sanschagrin F (2009). Newly introduced genomic prophage islands are critical determinants of in vivo competitiveness in the liverpool epidemic strain of pseudomonas aeruginosa. Genome Res.

[36] Visca P, Imperi F, Lamont IL (2007). Pyoverdine siderophores: from biogenesis to biosignificance. Trends Microbiol.

[37] Youard ZA, Wenner N, Reimmann C (2011). Iron acquisition with the natural siderophore enantiomers pyochelin and enantio-pyochelin in pseudomonas species. BioMetals.

[38] Dumas Z, Ross-Gillespie A, Kümmerli R (2013). Switching between apparently redundant iron-uptake mechanisms benefits bacteria in changeable environments. Proc Biol Sci.

[39] Ankenbauer R, Sriyosachati S, Cox CD (1985). Effects of siderophores on the growth of pseudomonas aeruginosa in human serum and transferrin. Infect Immun.

[40] Cox CD, Adams P (1985). Siderophore activity of pyoverdin for pseudomonas aeruginosa. Infect Immun.

[41] Prince RW, Cox CD, Vasil ML (1993). Coordinate regulation of siderophore and exotoxin a production: molecular cloning and sequencing of the Pseudomonas aeruginosa fur gene. J Bacteriol.

[42] Leoni L, Orsi N, De Lorenzo V, Visca P (2000). Functional analysis of PvdS, an iron starvation sigma factor of pseudomonas aeruginosa. J Bacteriol.

[43] Ratledge C, Dover LG (2000). Iron metabolism in pathogenic bacteria. Annu Rev Microbiol.

[44] Jiricny N, Diggle SP, West SA, Evans BA, Ballantyne G, Ross-Gillespie A (2010). Fitness correlates with the extent of cheating in a bacterium. J Evol Biol.

[45] Harrison F (2013). Dynamic social behaviour in a bacterium: pseudomonas aeruginosa partially compensates for siderophore loss to cheats. J Evol Biol.

[46] Reszka KJ, O’Malley Y, McCormick ML, Denning GM, Britigan BE (2004). Oxidation of pyocyanin, a cytotoxic product from pseudomonas aeruginosa, by microperoxidase 11 and hydrogen peroxide. Free Radic Biol Med.

[47] Kessler E, Safrin M, Olson JC, Ohman DE (1993). Secreted LasA of pseudomonas aeruginosa is a staphylolytic protease. J Biol Chem.

[48] R Development Core Team 2012. R: a language and environment for statistical computing. Vienna: R Foundation for Statistical Computing; 2012. http://www.R-project.org.

