# Supplementary figures and images for: High virulence sub-populations in Pseudomonas aeruginosa long-term cystic fibrosis airway infections
Source: BMC Microbiol. 2017 Feb 3;17:30. doi: 10.1186/s12866-017-0941-6 (PMC5291983; doi:10.1186/s12866-017-0941-6)

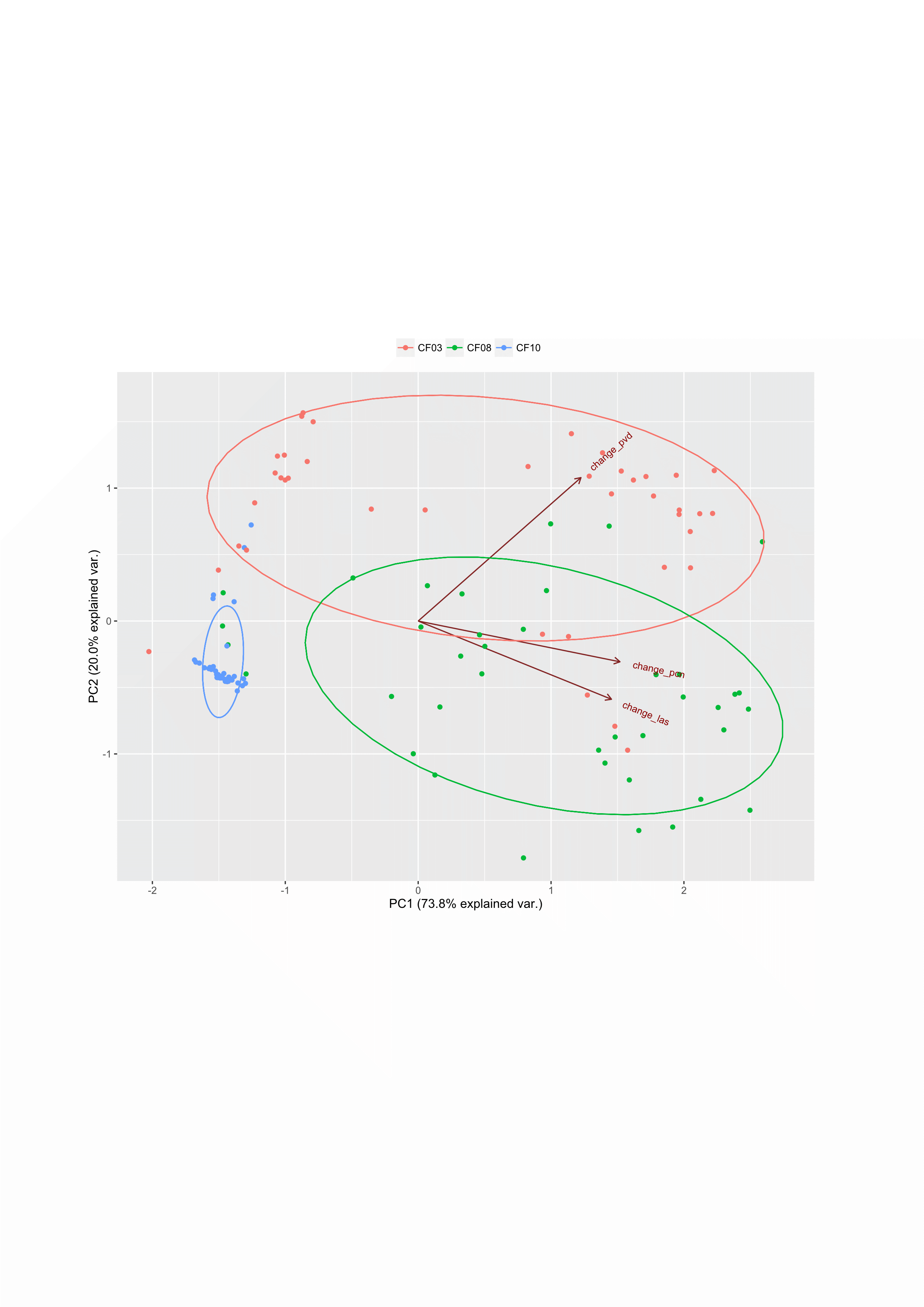

Supplement: Additional file 1: Figure S1. — Results of principal component analysis whereby all 188 isolates were pooled to investigate associations among production of these secretions. Overall, isolates typically either upregulated all three secretions, or produced very little of any (PC1 explains 73% variance). (GIF 368 kb) [file 12866_2017_941_MOESM1_ESM.gif]

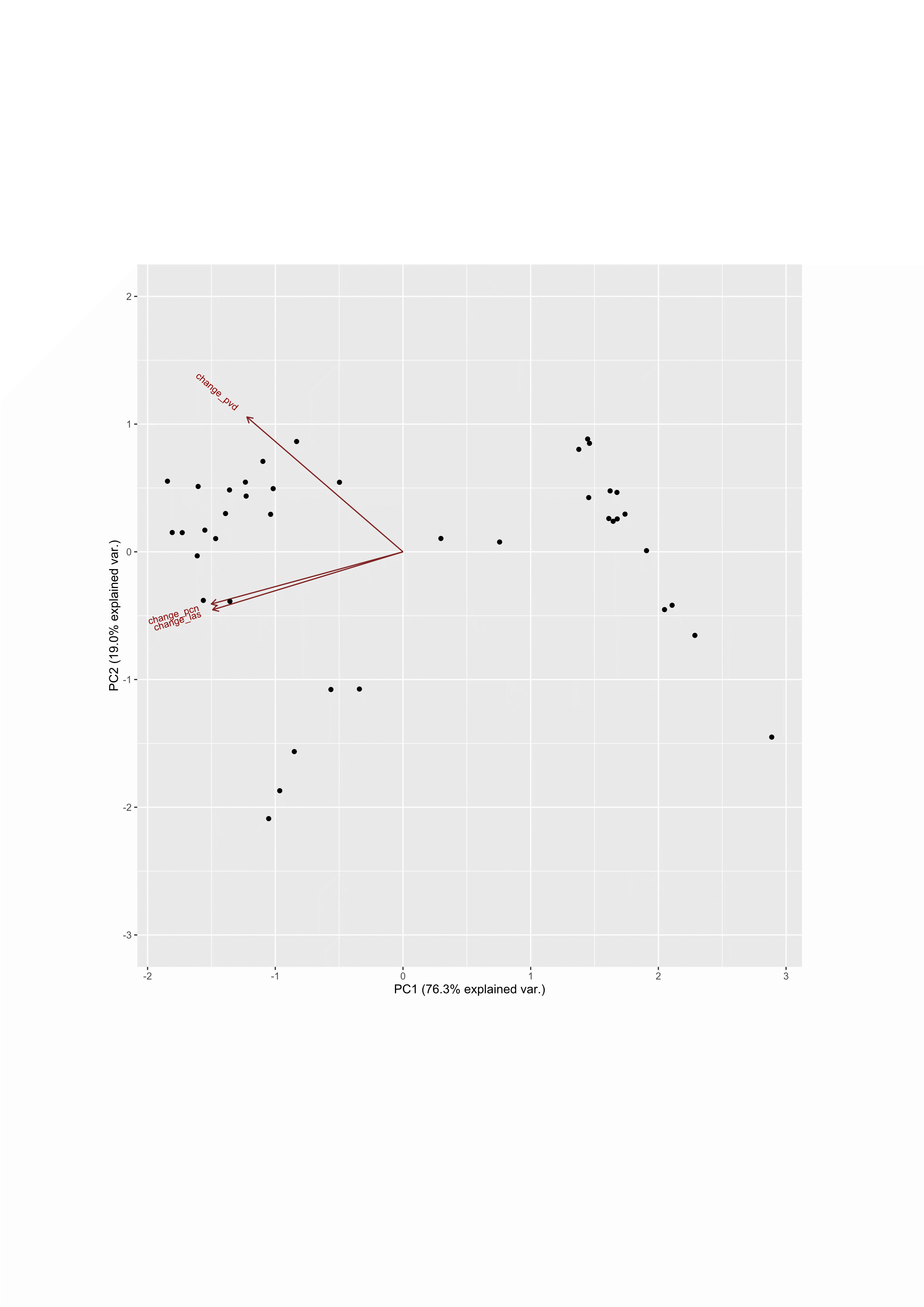

Supplement: Additional file 2: Figure S2. — Results of principal component analysis using phenotypic measurements from 40 isolates originating from patient CF03, showing that all three secretions were positively correlated, with the strongest association between LasA protease and pyocyanin (PC1, 76.3% variance). (GIF 194 kb) [file 12866_2017_941_MOESM2_ESM.gif]

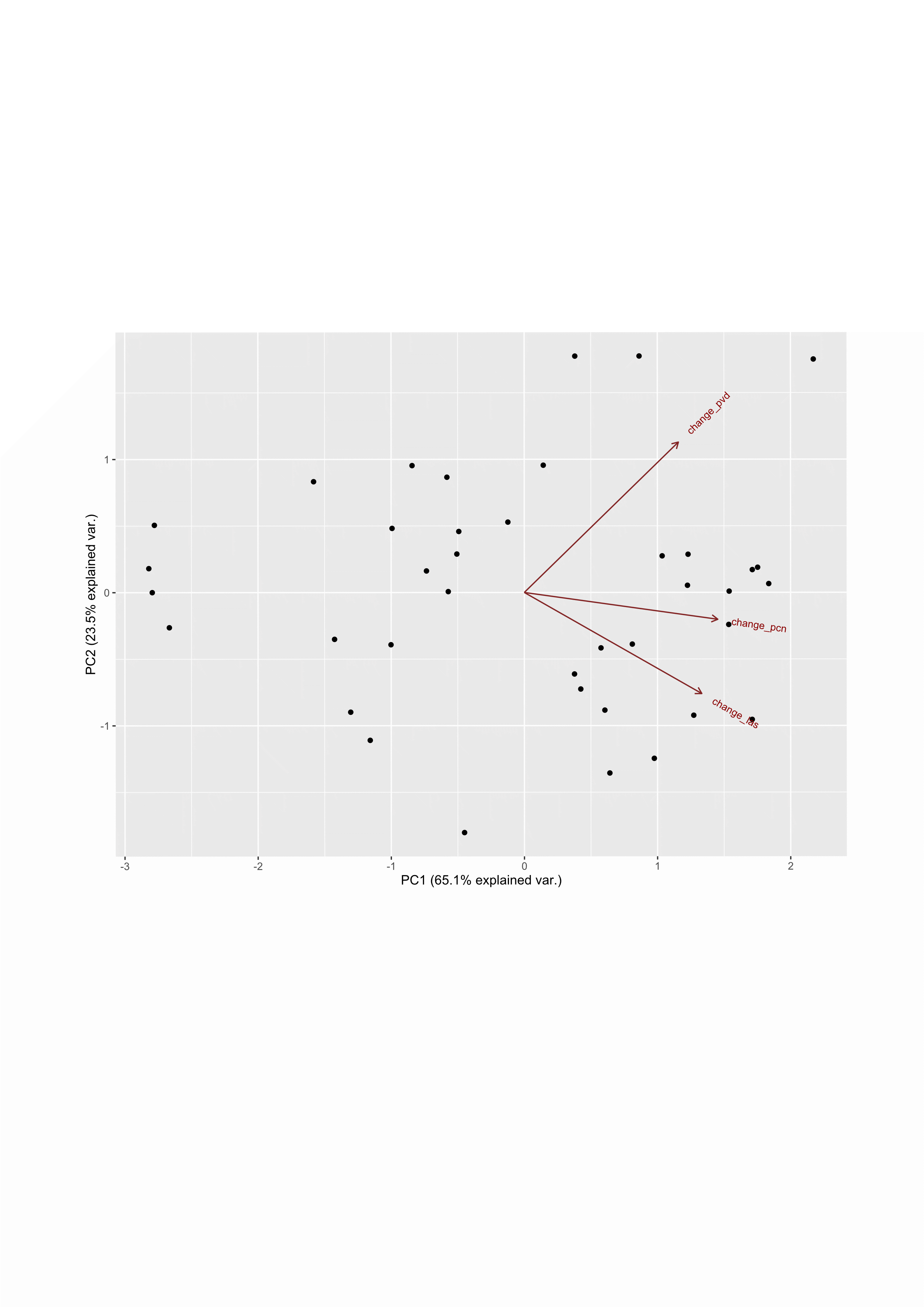

Supplement: Additional file 3: Figure S3. — Results of principal component analysis using phenotypic measurements from 39 isolates originating from patient CF08, showing that all three secretions were positively correlated, with the strongest association between LasA protease and pyocyanin (PC1, 65.1% variance). (GIF 212 kb) [file 12866_2017_941_MOESM3_ESM.gif]

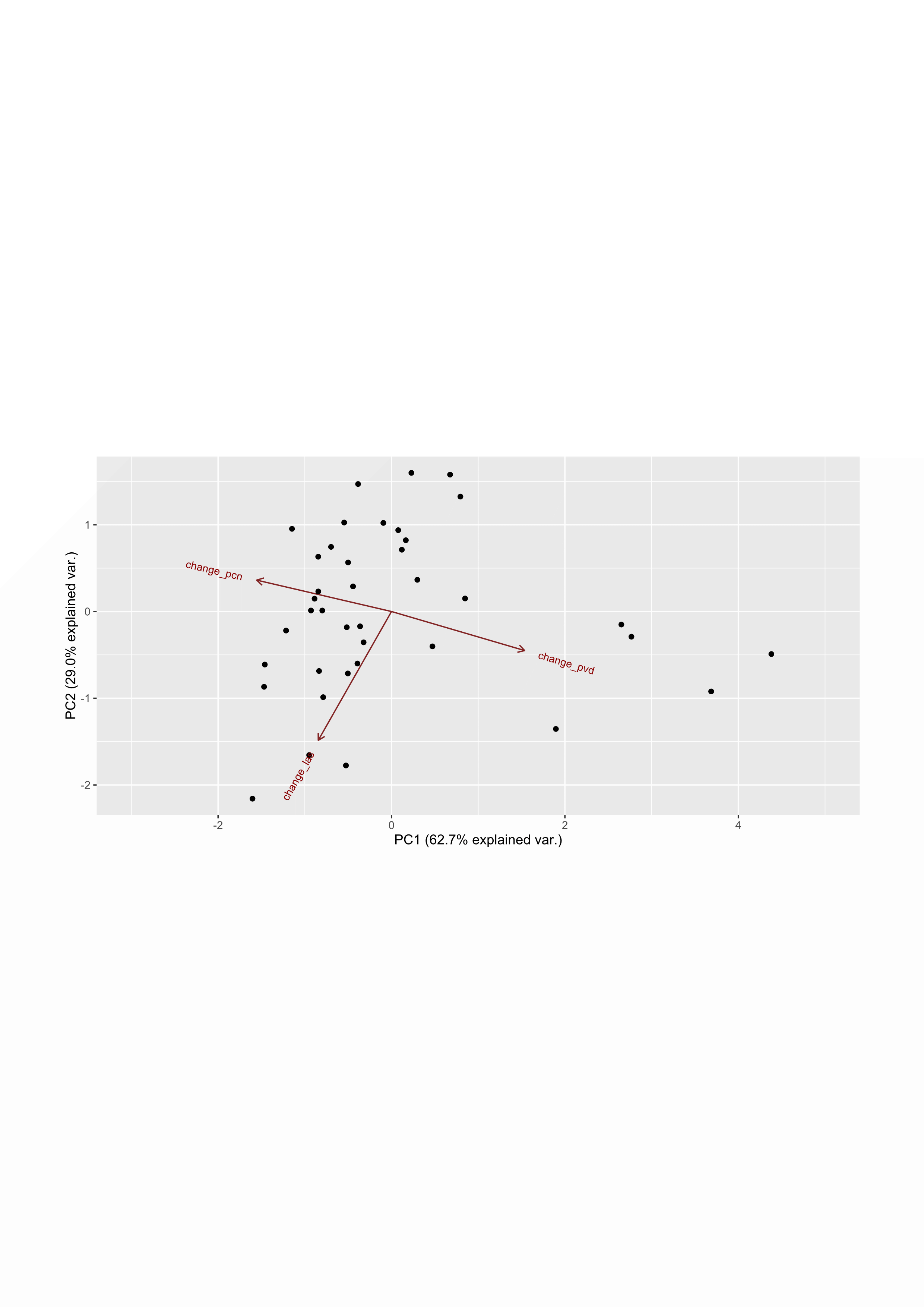

Supplement: Additional file 4: Figure S4. — Results of principal component analysis using phenotypic measurements from 39 isolates originating from patient CF10, revealing a negative correlation between pyocyanin and pyoverdine (PC1, 62.7% variance). (GIF 79 kb) [file 12866_2017_941_MOESM4_ESM.gif]
